# Air pollution increases gastroesophageal reflux disease risk: evidence from a prospective cohort study

**DOI:** 10.3389/fpubh.2025.1620411

**Published:** 2025-08-06

**Authors:** Yan Ran, Jian Lei, Lianli Wang, Laifu Li, Fangchen Ye, Lin Mei, Zhuoya Sun, Jiamiao Chen, Fei Dai

**Affiliations:** ^1^Department of Gastroenterology, The Second Affiliated Hospital of Xi'an Jiaotong University, Xi'an, Shaanxi, China; ^2^Key Laboratory of Environment and Genes Releated to Diseases, Department of Occupational and Environmental Health, Ministry of Education, School of Public Health, Xi'an Jiaotong University Health Science Center, Xi'an, Shaanxi, China

**Keywords:** gastroesophageal reflux disease, air pollution, particulate matter, gaseous pollutants, prospective cohort study

## Abstract

**Background:**

Gastroesophageal reflux disease (GERD) is one of the most prevalent gastrointestinal disorders with uncertain etiology and high prevalence. Ambient air pollution has been linked to gastrointestinal diseases, but the impact of long-term air pollution exposure on GERD incidence is still unclear.

**Methods:**

We performed a cohort study using the UK Biobank database. Annual mean concentrations of air pollutants, including PM_10_, PM_2.5–10_, PM_2.5_, NO_X_, and NO_2_, were obtained from the ESCAPE study using the land use regression model. The Cox proportional hazard regression model was employed to estimate the percentage change of GERD incidence risk related to long-term air pollutant exposures. We further explored the exposure-response relationship curves and identified the vulnerable populations.

**Results:**

During a follow-up period of 14.1 ± 2.4 years, a total of 32,413 (11.2%) individuals were diagnosed with GERD among 289,387 participants. We estimated that each interquartile range increase in PM_10_, PM_2.5–10_, PM_2.5_, NO_X_, NO_2,_ and NO was associated with 1.69, 1.29, 3.57, 2.08, 1.93, and 2.28% higher incidence risks of GERD, respectively. Almost linear exposure-response curves were observed, particularly for GERD without esophagitis. The females, middle-aged, overweight, White ethnicity, and higher socioeconomic status individuals were more vulnerable to GERD when exposed to air pollutants.

**Conclusion:**

This study provided robust evidence supporting the association between long-term exposure to air pollutants and increased risk of GERD incidence. Our research revealed that exposure to both particulate matter and gaseous pollutants was associated with a higher risk of GERD, especially for GERD without esophagitis.

## 1 Introduction

Gastroesophageal reflux disease (GERD) is one of the most prevalent gastrointestinal disorders, which is defined as the movement of stomach contents into the esophagus or mouth, causing troublesome symptoms or complications with heartburn and reflux as the most typical symptoms (1, 2). According to estimates from a global epidemiological study, approximately 13.3% of the population worldwide suffered from heartburn or reflux symptoms at least once a week, with prevalence varying from 2.5 to 51.2% across different countries (3). Worse still, the number continues to rise (4). Due to its high prevalence and chronicity, GERD impairs the patient’s quality of life seriously and contributes to substantial economic and medical burdens. GERD has been identified to increase the risk of esophageal strictures and esophageal carcinoma (5), which can be life-threatening. Reflux exposure, epithelial resistance, inflammation, motility disorder, and visceral hypersensitivity were all involved in the complicated pathogenesis of GERD (6, 7).

Ambient air pollution is a prominent environmental risk factor for various diseases. Particulate matter has been the leading contributor to the Global Burden of Disease Study 2021 (GBD 2021) (8). Inhalable particulate matter (PM_10_) could be mainly classified into coarse particulate matter (PM_2.5–10_) and fine particulate matter (PM_2.5_) (9). According to the Integrated Science Assessment (ISA), approximately 71% of the particulate matter deposited in the nose is subsequently transported to the gastrointestinal tract (9), underlining the significance of the gastrointestinal exposure pathway for air pollutants. Nitrogen oxides (NO_X_) are one of the typical types of gaseous pollutants, consisting of nitrogen dioxide (NO_2_) and nitric oxide (NO). NO_X_ is mainly sourced from vehicle emissions, industrial processes, and fuel combustion, and has specific adverse health effects (10, 11). It is predicted that NO_X_ is emitted into the atmosphere predominantly as NO (more than 90%) (10), which is a highly reactive free radical as a noxious air pollutant (12). While previous research has largely focused on respiratory and cardiovascular diseases, only limited studies have examined its role in gastrointestinal disorders (13–15). In particular, the potential association between long-term exposure to air pollutants and the risk of GERD incidence has not been investigated.

To address existing knowledge gaps, our study aimed to evaluate the impact of long-term particulate matter (PM_10_, PM_2.5–10_, and PM_2.5_) and gaseous pollutants (NO_X_, NO_2_, and NO) exposures on incident GERD as well as its subtypes (GERD without esophagitis and GERD with esophagitis). Furthermore, we sought to identify populations that may be more susceptible to the association between air pollution and incident GERD.

## 2 Methods

### 2.1 Study population

We performed this cohort study using data from the UK Biobank database, which is a population-based longitudinal study of around 0.5 million participants aged 40 to 69 years enrolled from 22 health assessment centers in the UK between 2006 and 2010 (16). During baseline assessment, a wide range of health-related information was collected from touchscreen questionnaires, physical measurements, and biological samples. The UK Biobank study was approved by the North West Multicenter Research Ethics Committee, and all participants provided written informed consent before data collection. Our research utilized data from this approved project (application ID: 99732) within the UK Biobank.

In this study, we aimed to investigate the associations between air pollutant exposures and the risk of GERD incidence. Among a total of 502,271 participants, individuals with a diagnosis of cancer (*n* = 38,647) or GERD (*n* = 4,326) at baseline were excluded. To reduce the impact of other related factors, we excluded participants with GERD-related conditions (including esophagitis, other diseases of the esophagus, peptic ulcer, gastritis and duodenitis, other diseases of stomach and duodenum) (*n* = 602) or take acid inhibitors medicine (proton pump inhibitors or H_2_ antagonist) (*n* = 139,678). Participants with incomplete socioeconomic data or air pollutants data (*n* = 29,631) were also excluded. Finally, a total of 289,387 participants were included in the statistical analysis (Supplementary Figure S1).

### 2.2 Assessment of exposure

The ambient air pollutants examined in this study included PM_10_, PM_2.5–10_, PM_2.5_, NO_X_, NO_2_, and NO. The land use regression (LUR) model, developed as part of the European Study of Cohorts for Air Pollution Effects (ESCAPE) project, was employed to estimate the annual mean air pollutant concentrations in 2010 (17, 18). Annual average concentrations of air pollutants were evaluated using pollutant-specific LUR models, which utilize predictor variables derived from the Geographic Information System and linked to participants’ residential addresses obtained from baseline information collection. The exposure data of PM_2.5–10_, PM_2.5_, and NO_X_ were collected in 2010, while annual concentration data of PM_10_ and NO_2_ were available for several years (2007 and 2010 for PM_10_; 2005–2007, and 2010 for NO_2_). Following the official guidelines provided by the UK Biobank, data from various air pollution models should not be averaged. Consequently, for our analysis, we utilized the air pollution data for 2010 from the ESCAPE project to represent long-term exposure, consistent with methodologies employed in related prior research (19). NO_X_ refers specifically to the sum of nitrogen dioxide (NO_2_) and nitric oxide (NO). We estimated the NO concentration by subtracting the concentration of NO_2_ from NO_X_.

### 2.3 Assessment of outcome

The outcome was defined as the first occurrence of a GERD diagnosis, encoded as K21 according to the International Classification of Diseases 10th Revision (ICD-10). Based on ICD-10, the first occurrence of K21 (GERD), including K21.0 (GERD with esophagitis) and K21.9 (GERD without esophagitis), was considered the outcome of this study. The follow-up period extended from the date of baseline assessment to the date of GERD diagnosis. For individuals who did not develop GERD, the endpoint was defined as the earliest of the following events: death, loss to follow-up, or the end of the study (May 2024).

### 2.4 Assessment of covariates

The covariates related to air pollution and/or GERD were initially determined by reviews of relevant studies (1, 3, 13), including age, sex, body mass index (BMI), ethnicity, education level, dietary habits, physical activity, smoking status, alcohol consumption, mental health disorders, the Townsend Deprivation Index (TDI), and assessment centers. To determine which covariates should be adjusted in our model, we introduced a graphical tool called directed acyclic graphs (DAGs). DAGs are widely used to identify confounding variables that require adjusting to estimate causal effects (20). The DAGitty online tool^1^ was utilized to construct a DAG for our study. We identified six confounders—age, sex, ethnicity, education level, the TDI, and assessment centers—which required adjustment in the main model (Supplementary Figure S2). For more detailed information on methodology, please refer to the Supplementary Methods.

### 2.5 Statistical analysis

In the present study, the Cox proportional hazard regression model was applied to estimate the association between long-term exposure to air pollutants and GERD incidence. Results are expressed as the percentage change (%) in risk, which is calculated based on the hazard ratios (HRs) after adjusting for potential confounders (age, sex, ethnicity, education level, the TDI, and assessment centers). Specifically, the percentage change was calculated as [(HR − 1)/1] × 100%, representing the relative change in GERD risk associated with per interquartile range (IQR) increase in air pollutant. Statistical significance is determined based on whether the 95% CI crosses zero for the percentage change (equivalent to crossing one for the HR). The proportional hazard assumption was tested using Schoenfeld residuals and was not violated.

We used single-pollutant models (including each pollutant separately) to estimate the air pollution-related GERD risk. The linear exposure-response relationship between each air pollutant exposure and GERD incidence risk was assessed by calculating the trend *p*-values for each pollutant exposure. Furthermore, we investigated the exposure-response association using a natural cubic spline with 2 degrees of freedom.

Subgroup analyses were conducted by sex (male vs. female), age [<60 years (middle-aged) vs. ≥60 years (older adults)], BMI (< 25 vs. ≥ 25 kg/m^2^), ethnicity (White vs. others), education level (low vs. high), TDI (low SES vs. high SES), and assessment centers (England vs. others) to identify the vulnerable populations. The statistical significance of the difference between strata was assessed using a two-sample *Z* test, applied according to the following formula:


$$Z=\frac{Q_{1}-Q_{2}}{\sqrt{SE_{1}^{2}+SE_{2}^{2}}}$$

where Q_1_ and Q_2_ are the strata-specific regression coefficients, and SE_1_ and SE_2_ are the corresponding standard errors (21).

Additionally, we conducted a series of sensitivity analyses to assess the robustness and reliability of our findings: (1) additional adjustment for behavioral factors (BMI, diet, physical activity, smoking, alcohol, and mental health disorders); (2) excluding cases diagnosed within the first 1–3 years of follow-up to minimize potential reverse causation; (3) restricting the analysis to participants residing at their current address for at least 10 years to reduce exposure misclassification; and (4) using transport accidents (ICD: V01–V99) as a negative control outcome to evaluate unmeasured confounding.

Statistical analyses for this study were performed using R (version 4.2.1; R Foundation for Statistical Computing). The Cox proportional hazards regression model was implemented via the *survival* package. The two-sided *p*-values < 0.05 were statistically significant.

## 3 Results

### 3.1 Baseline characteristics

Table 1 displays the baseline characteristics of participants in this study. Of 289,387 participants, the mean (SD) age was 57.2 ± 8.0 years, and more than half were females (56.2%). During a follow-up period of 14.1 ± 2.4 years, 32,413 (11.2%) incident cases of GERD were identified, including 27,669 cases of GERD without esophagitis (K21.9) and 8,897 cases of GERD with esophagitis (K21.0). The concentration of pollutants (mean, SD, minimum, IQR, percentile, and maximum) is presented in Table 2. The mean estimates of annual average concentrations for PM_10_, PM_2.5–10_, PM_2.5_, NO_X_, NO_2_ and NO were 16.23, 6.42, 9.99, 43.99, 26.60, and 17.39 μg/m^3^, respectively (Table 2), which exceeded the threshold recommended by the World Health Organization Global Air Quality Guidelines (AQG 2021: PM_10_, 15 μg/m^3^; PM_2.5_, 5 μg/m^3^; NO_2_, 10 μg/m^3^) (22). Spearman correlation analysis among pollutants showed correlation coefficients below 0.8 (Supplementary Table S1).

**Table 1:** Baseline characteristics of the included participants.

	GERD	GERD without esophagitis	GERD with esophagitis	Total population
Number of participants (*N*, %)	32,413	27,669	8,897	289,387
Follow-up period (years)	7.9 ± 3.7	8.2 ± 3.6	7.2 ± 3.9	14.1 ± 2.4
Age at recruitment	58.8 ± 7.4	58.9 ± 7.4	58.7 ± 7.4	57.2 ± 8.0
Sex				
Male	13,525 (41.7)	11,285 (40.8)	4,065 (45.7)	126,719 (43.8)
Female	18,888 (58.3)	16,384 (59.2)	4,832 (54.3)	162,668 (56.2)
Age (years)				
<60	14,624 (45.1)	12,265 (44.3)	4,089 (46.0)	152,850 (52.8)
≥60	17,789 (54.9)	15,404 (55.7)	4,808 (54.0)	136,537 (47.2)
Body mass index (kg/m^2^)				
<25	7,697 (23.7)	6,384 (23.1)	2,168 (24.4)	87,561 (30.3)
≥25	24,568 (75.8)	21,147 (76.4)	6,696 (75.3)	200,366 (69.2)
Missing data	148 (0.5)	138 (0.5)	33 (0.4)	1,460 (0.5)
Ethnic background				
White	29,815 (92.0)	25,422 (91.9)	8,209 (92.3)	263,216 (91.0)
Mixed	1,096 (3.4)	939 (3.4)	313 (3.5)	10,508 (3.6)
Asian or Asian British	904 (2.8)	767 (2.8)	246 (2.8)	9,590 (3.3)
Black or Black British	173 (0.5)	157 (0.6)	43 (0.5)	1,686 (0.6)
Others	425 (1.3)	384 (1.4)	86 (1.0)	4,387 (1.5)
Education level^1^				
Low	16,477 (50.8)	13,971 (50.5)	4,561 (51.3)	146,012 (50.5)
High	7,287 (22.5)	6,056 (21.9)	2,046 (23.0)	85,593 (29.6)
Missing data	8,649 (26.7)	7,642 (27.6)	2,290 (25.7)	57,782 (20.0)
Townsend Deprivation Index^2^				
Low SES	16,217 (50.0)	13,824 (50.0)	4,462 (50.2)	145,073 (50.1)
High SES	16,196 (50.0)	13,845 (50.0)	4,435 (49.8)	144,314 (49.9)
Smoking status				
Never	15,972 (49.3)	13,629 (49.3)	4,328 (48.6)	153,812 (53.2)
Previous smoking	12,851 (39.7)	11,006 (39.8)	3,509 (39.4)	104,614 (36.2)
Current smoking	3,408 (10.5)	2,874 (10.4)	1,012 (11.4)	29,770 (10.3)
Missing data	182 (0.6)	160 (0.6)	48 (0.5)	1,191 (0.4)
Drinking status				
Never	1,768 (5.5)	1,589 (5.7)	427 (4.8)	13,653 (4.7)
Previous drinking	1,657 (5.1)	1,490 (5.4)	431 (4.8)	11,640 (4.0)
Current drinking	28,933 (89.3)	24,543 (88.7)	8,023 (90.2)	263,700 (91.1)
Missing data	55 (0.2)	47 (0.2)	16 (0.2)	394 (0.1)
Diet^3^				
Healthy	28,525 (88.0)	24,345 (88.0)	7,787 (87.5)	256,512 (88.6)
Unhealthy	3,888 (12.0)	3,324 (12.0)	1,110 (12.5)	32,875 (11.4)
Physical activity^4^				
Low	5,040 (15.5)	4,284 (15.5)	1,380 (15.5)	42,999 (14.9)
Moderate	9,416 (29.1)	7,971 (28.8)	2,600 (29.2)	89,668 (31.0)
High	9,134 (28.2)	7,731 (27.9)	2,595 (29.2)	87,938 (30.4)
Missing data	8,823 (27.2)	7,683 (27.8)	2,322 (26.1)	68,782 (23.8)
Mental disorders^5^				
Yes	5,440 (16.8)	4,767 (17.2)	1,485 (16.7)	37,539 (13.0)
No	26,781 (82.6)	22,732 (82.2)	7,364 (82.8)	250,338 (86.5)
Missing data	192 (0.6)	170 (0.6)	48 (0.5)	1,510 (0.5)

^1^Education level was dichotomized as high (college or university degree) and low (high school or below).

^2^High SES was defined as Townsend Deprivation Index < −2.17 (median value) while low SES was defined as Townsend Deprivation Index ≥ −2.17.

^3^Healthy diet was defined as at least two of the healthy foods (fruit and vegetable intake: >4.5 pieces or servings a week; fish intake: >2 per week; meat intake: processed meat ≤ 2 per week and red meat ≤ 5 per week), otherwise unhealthy.

^4^Physical activity was categorized as low, moderate, and high based on the International Physical Activity Questionnaire (IPAQ).

^5^Mental disorders were assessed according to the touchscreen question (“Have you ever seen a psychiatrist for nerves, anxiety, tension, or depression?”).

SES, socioeconomic status.

**Table 2:** Descriptive statistics for the annual concentrations of ambient air pollutants in the United Kingdom in 2010.

Air pollutants	Mean	SD	IQR	Min	Percentile					Max
					P1	P25	P50	P75	P99	
Particulate matter										
PM_10_	16.23	1.89	1.75	11.78	11.98	15.25	16.02	17.00	21.47	30.65
PM_2.5–10_	6.42	0.90	0.79	5.57	5.59	5.84	6.11	6.63	9.19	12.82
PM_2.5_	9.99	1.06	1.27	8.17	8.17	9.29	9.93	10.56	13.17	21.31
Gaseous pollutants										
NO_X_	43.99	15.55	16.43	19.74	20.61	34.25	42.20	50.68	95.63	265.94
NO_2_	26.60	7.57	9.73	12.93	13.02	21.38	26.07	31.11	47.78	108.49
NO	17.39	9.07	9.01	0.00	5.48	11.69	16.05	20.70	49.68	160.06

Air pollutant concentrations are measured in μg/m^3^.

PM_10_, inhalable particulate matter; PM_2.5–10_, coarse particulate matter; PM_2.5_, fine particulate matter; NO_X_, nitrogen oxides; NO_2_, nitrogen dioxide; NO, nitric oxide. SD, standard deviation; IQR, interquartile range; Min, minimum; P, Percentile; Max, maximum.

### 3.2 Associations of air pollutants with incident GERD

**Figure 1:**
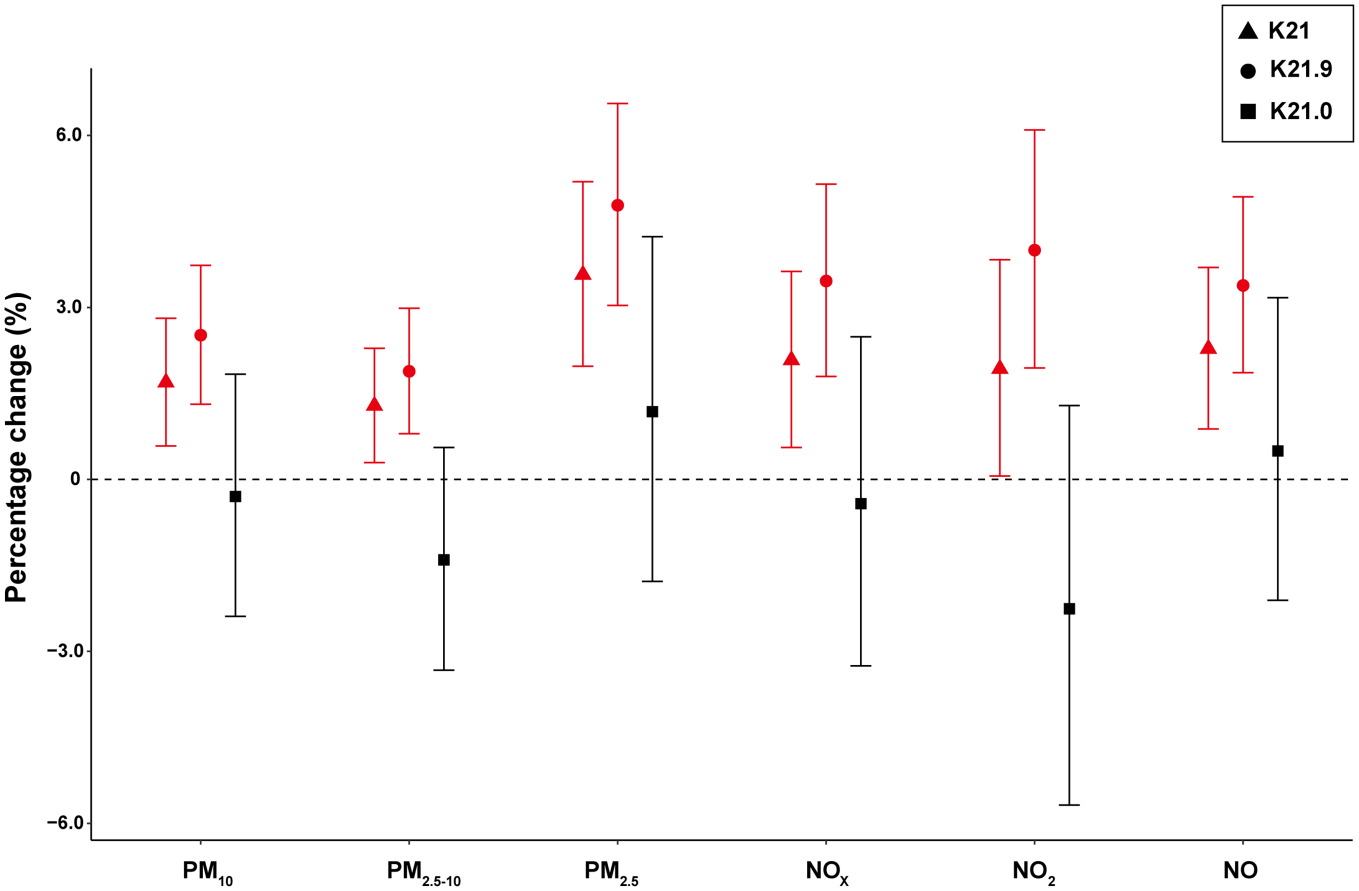
The estimated association between percentage change (%) in GERD incidence risk associated with each IQR in air pollutants. The interquartile range (IQR) values for PM_10_, PM_2.5–10_, PM_2.5_, NO_X_, NO_2_, and NO were 1.75, 0.79, 1.27, 16.43, 9.73, and 9.01 μg/m^3^, respectively. The model was adjusted for age, sex, ethnicity, education level, Townsend deprivation index, and assessment centers. Abbreviation: PM_10_, inhalable particulate matter; PM_2.5–10_, coarse particulate matter; PM_2.5_, fine particulate matter; NO_X_, nitrogen oxides; NO_2_, nitrogen dioxide; NO, nitric oxide; K21, gastroesophageal reflux disease (GERD); K21.9, GERD without esophagitis; K21.0, GERD with esophagitis; IQR, interquartile range.

Assessing by Cox proportional hazard regression models, significantly positive associations of particulate matter (PM_10_, PM_2.5–10_, and PM_2.5_) and gaseous pollutants (NO_X_, NO_2_, and NO) with incident GERD were observed (Table 3 and Figure 1). Overall, for each IQR increase in long-term PM_10_, PM_2.5–10_, PM_2.5_, NO_X_, NO_2_, and NO exposure, the risk of GERD increased 1.69% (95% CI: 0.58, 2.81%), 1.29% (95% CI: 0.29, 2.29%), 3.57% (95% CI: 1.97, 5.19%), 2.08% (95% CI: 0.56, 3.63%), 1.93% (95% CI: 0.06, 3.83%), and 2.28% (0.88, 3.70%), individually. When stratified by GERD subtype, the positive associations were predominantly observed for GERD without esophagitis (K21.9), with no significant association for GERD with esophagitis (K21.0).

**Table 3:** Percentage change in the risk of incident GERD per IQR increase in air pollutants.

Air pollutants	Particulate matter			Gaseous pollutants		
	PM_10_	PM_2.5–10_	PM_2.5_	NO_X_	NO_2_	NO
K21	1.69 (0.58, 2.81)	1.29 (0.29, 2.29)	3.57 (1.97, 5.19)	2.08 (0.56, 3.63)	1.93 (0.06, 3.83)	2.28 (0.88, 3.70)
K21.9	2.52 (1.31, 3.73)	1.89 (0.80, 2.99)	4.78 (3.03, 6.56)	3.46 (1.80, 5.15)	4.00 (1.94, 6.10)	3.38 (1.86, 4.93)
K21.0	−0.30 (−2.39, 1.83)	−1.40 (−3.33, 0.56)	1.18 (−1.78, 4.23)	−0.42 (−3.25, 2.49)	−2.26 (−5.68, 1.29)	0.50 (−2.11, 3.17)

Results are expressed as the percentage change (%) in risk, and percentage signs (%) are omitted for clarity. The interquartile range (IQR) values for PM_10_, PM_2.5–10_, PM_2.5_, NO_X_, NO_2_, and NO were 1.75, 0.79, 1.27, 16.43, 9.73, and 9.01 μg/m^3^, respectively. The model was adjusted for age, sex, ethnicity, education level, Townsend deprivation index, and assessment centers.

PM_10_, inhalable particulate matter; PM_2.5–10_, coarse particulate matter; PM_2.5_, fine particulate matter; NO_X_, nitrogen oxides; NO_2_, nitrogen dioxide; NO, nitric oxide; K21, gastroesophageal reflux disease (GERD); K21.9, GERD without esophagitis; K21.0, GERD with esophagitis; IQR, interquartile range.

**Figure 2:**
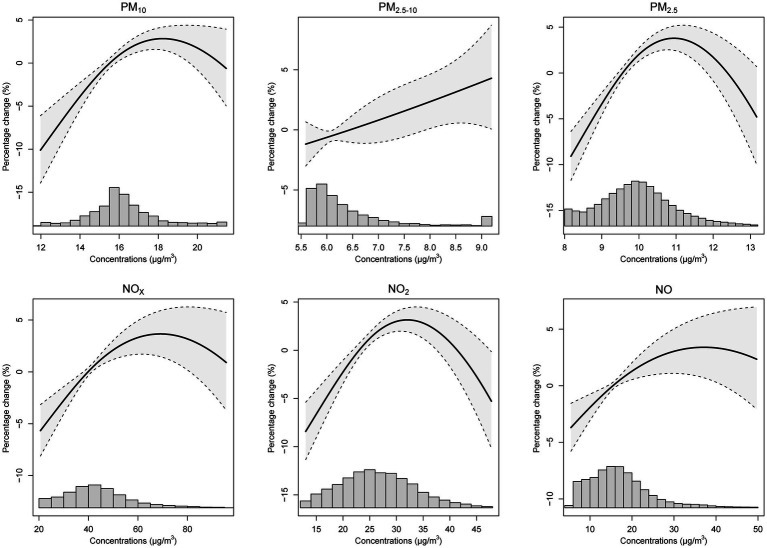
The exposure and response curves of long-term ambient air pollutant exposures and the percentage change of GERD (K21) incident risk. Note: the model was adjusted for age, sex, ethnicity, education level, Townsend deprivation index, and assessment centers. PM_10_, inhalable particulate matter; PM_2.5–10_, coarse particulate matter; PM_2.5_, fine particulate matter; NO_X_, nitrogen oxides; NO_2_, nitrogen dioxide; NO, nitric oxide; GERD, gastroesophageal reflux disease.

Furthermore, exposure-response curves (Figure 2) demonstrated an almost linear relationship between PM_2.5–10_ and the incidence risks of GERD (K21). The risk of GERD followed approximately linearly increasing associations with other pollutants (PM_10_, PM_2.5_, NO_X_, NO_2_, and NO) at lower exposure levels, with subtle downward trends at higher exposures. Similar trends were shown between air pollutants and GERD without esophagitis (K21.9) (Supplementary Figure S3), while there was no linear exposure-response relationship between air pollution and GERD with esophagitis (K21.0) (Supplementary Figure S4).

### 3.3 Subgroup and sensitivity analyses

Subgroup analyses revealed that the middle-aged (<60 years), White ethnicity, those with higher socioeconomic status (SES), and residents of England were more susceptible to particulate matter exposure. No statistically significant effect was observed when stratified by sex, BMI, or education level. For gaseous pollutants, increased risk was observed among females, the middle-aged (<60 years), overweight (BMI ≥ 25 kg/m^2^) individuals, and those of White ethnicity and higher SES. No statistically significant differences were observed when stratified by education level or assessment center (Supplementary Tables S2, S3).

Sensitivity analyses verified the robustness of the associations observed between air pollutants and GERD in the main model analysis. When adding other covariates to the main model, excluding participants whose GERD diagnosis occurred in the 1/2/3 years of follow-up, or restricting the analysis to participants who lived in their current address for at least 10 years, the association between air pollutants (except for NO_2_) exposure with GERD incidence did not significantly change (Supplementary Table S4). In addition, we did not observe statistically significant associations between air pollutant exposures and the risk of traffic accidents (negative control group) (Supplementary Table S5).

## 4 Discussion

In this large-scale and long-term prospective cohort study, we estimated a robust association between several air pollutants and the risk of GERD. Our findings indicated that long-term exposure to both particulate matter (PM_10_, PM_2.5–10_, and PM_2.5_) and gaseous pollutants (NO_X_, NO_2_, and NO) is associated with an increased risk of GERD incidence, particularly for GERD without esophagitis (K21.9). We observed almost linear exposure-response curves for the association between long-term exposure to air pollutants and the risk of GERD. Subgroup analyses revealed a stronger association with particulate matter among individuals under 60 years old, while females, individuals under 60 years old, and the overweight individuals (BMI ≥ 25 kg/m^2^) were more sensitive to gaseous pollutants.

Most previous research has concentrated on the effects of air pollution on respiratory and cardiovascular diseases (23–25), with relatively limited attention to the associations between air pollution and gastrointestinal diseases. Several previous epidemiological studies have revealed associations between air pollutant exposure and gastrointestinal diseases. In accordance with previous research examining the relationship between long-term PM_2.5_ exposure and the risk of esophageal cancer incidence (13), our study also observed nearly linear exposure-response curves for the association between long-term PM_2.5_ and PM_10_ exposure and GERD. Although it is well established that GERD is a major risk factor for esophageal cancer (5), current evidence is insufficient to confirm whether air pollution indirectly increases the risk of esophageal cancer by promoting GERD progression. Further studies are needed to confirm this potential linkage. Only a few studies have investigated the potential association between air pollution and GERD. One study from Korea found that GERD-related medical utilization increased with the levels of PM_2.5_ and carbon monoxide (26), which only reflected healthcare utilization patterns rather than the incidence risk of GERD. Furthermore, the study did not differentiate GERD subtypes. Another cohort study examined the relationships of air pollutants and the risk of multiple gastrointestinal diseases (27), which included GERD in their investigation, and found an association between PM_2.5_ exposure and GERD. In contrast to these two studies, our study offers a more comprehensive and disease-specific perspective by analyzing the associations between multiple air pollutants and GERD incidence, including distinct analyses by GERD subtypes. Moreover, we conducted comprehensive subgroup analyses to identify vulnerable populations and estimated exposure-response relationships, which provide novel insights into pollutant-specific and subtype-specific risks of GERD.

Additionally, we observed a stronger association between air pollution and GERD without esophagitis (K21.9) compared to GERD with esophagitis (K21.0). This difference is more likely attributable to the different pathophysiological mechanisms underlying these two subtypes. GERD with esophagitis is characterized by visible mucosal injury on endoscopy and is primarily associated with prolonged acid exposure and overt epithelial damage (28), which may be less directly influenced by pollutant-induced pathways. In contrast, GERD without esophagitis lacks macroscopic mucosal erosion and involves impaired mucosal resistance, increased epithelial permeability, and enhanced visceral hypersensitivity (29, 30), which could be more susceptible to pollution-induced oxidative stress and inflammatory responses.

The associations between air pollutant exposures and GERD present a biological mechanism of rationality. In addition to the respiratory tract, the gastrointestinal tract is another important exposure route for air pollutants (9). Particulate matter can enter the gastrointestinal tract through multiple pathways, including ingestion of contaminated food and water, mucociliary clearance from the respiratory tract, and the systemic blood circulation (31–33). Besides, gaseous pollutants might also affect the digestive tract through swallowed air. Although NO_X_ itself is unlikely to enter the bloodstream directly, its various reaction products may migrate into the blood and spread to other tissues or organs (10). All the above indicate that the gastrointestinal tract serves as an important pathway for exposure to air pollutants.

Although direct evidence linking air pollution to GERD remains limited, existing studies have demonstrated that air pollutants can induce oxidative stress, systemic inflammation, and epithelial barrier dysfunction in the gastrointestinal tract (34–37), which provides important biological plausibility for the observed associations in our study. Experimental studies indicated that exposure to particulate matter may enhance the generation of reactive oxygen species (ROS), cause damage to epithelial cells, and lead to the disruption and increased permeability of the gastrointestinal barrier (35). Furthermore, particulate matter exposure could activate immune and inflammatory responses in the gastrointestinal tract. It has been reported that exposure to particulate matter is associated with increased infiltration of inflammatory cells, heightened expression of inflammation-related genes (such as IL-1β, IL-6, and TNF-*α*), and exacerbation of mucosal inflammation in the colon (36, 37). Barrier disruption, immune and inflammation activation contributed to the pathogenesis of GERD, highlighting potential mechanisms that may underlie the association between particulate matter exposure and GERD. Regarding gaseous pollutants, this is the first cohort study to evaluate the association between ambient NO_X_ exposure and the risk of GERD, which revealed that both NO_2_ and NO exposures increase the risk of GERD without esophagitis (K21.9). The association between NO_X_ exposure and GERD is biologically plausible. NO_X_ can convert into various reactive nitrogen oxide species (RNOS) in the human body. RNOS at the human gastro-esophageal junction can damage the barrier function of the adjacent tissue by disrupting the tight junction (38). Studies suggested that the products of NO_2_, such as nitrite, may migrate into the blood and induce systemic inflammation and oxidative stress, providing a potential mechanism by which NO_2_ exposure could lead to health effects beyond the respiratory system (10). Additionally, swallowed NO_2_ might cause nitration of different compounds, including nitrate and nitrite in the stomach, which could induce the redox interplay and inflammatory response (14, 39). Previous studies have provided evidence that NO can inhibit esophageal motility, disrupt epithelial barrier function, exacerbate inflammation, and accelerate columnar transformation in the esophagus (39, 40), suggesting that NO could also contribute to GERD pathogenesis in addition to conventional causative factors. This evidence provided biological plausibility and enhances the reliability of our findings. While direct evidence remains limited, these mechanisms may provide important biological plausibility for the associations observed in our study. Further experimental and longitudinal studies are needed to confirm the causal relationships and to elucidate the exact role of air pollutants in the pathogenesis of GERD.

Our subgroup analyses indicated that the associations between air pollutants and GERD varied in different groups. Specifically, we observed that females displayed greater sensitivity, especially for gaseous pollutants. Several previous studies have reported increased risks among females (41, 42), which may partly be attributed to sex-related differences in susceptibility and the greater exposure to indoor air pollution, particularly from sources such as cooking (41). We also observed that the risk of GERD tended to be higher in middle-aged individuals (<60 years) when exposed to air pollutants. Younger populations tend to experience greater exposure to air pollution due to their increased frequency of outdoor activities compared to older individuals, which may be one of the reasons why the younger population is more vulnerable. Besides, we discovered stronger associations between air pollutants (especially NO_X_, NO_2_, and NO) and GERD in overweight individuals (BMI ≥ 25 kg/m^2^) than those with BMI < 25 kg/m^2^, which is consistent with other studies that air pollution exposure associated with overweight and obesity (43, 44). Additionally, we observed stronger associations in White ethnicity and those with higher SES. A previous study also found that GERD was more prevalent among individuals with higher SES (45). The differential vulnerability may be partially explained by SES-related lifestyle factors, such as dietary patterns and obesity. Moreover, individuals with higher SES are more likely to seek medical attention for GERD symptoms and undergo diagnostic testing, which may contribute to increased case detection.

Our study exhibits several significant advantages. First, it represents the first large-scale, population-based, and long-term cohort study revealing the impact of both particulate matter (PM_10_, PM_2.5–10_, and PM_2.5_) and gaseous pollutants (NO_X_, NO_2_, and NO) on different subtypes of GERD (including K21.9 and K21.0). The large sample size and long follow-up period greatly enhanced the statistical power. Second, we introduced a DAG to provide a comprehensive and transparent process for more appropriately selecting confounders, which boosts the scientificity of our study. Finally, we conducted several sensitivity analyses to verify the robustness of the main model, further improving the credibility of the results.

It is also important to acknowledge several limitations in our study. First, because of the limited time scale of exposure data available in the UK Biobank, we used the annual average concentration of air pollutants for the year 2010 to represent long-term exposure, which is in accordance with methodologies employed in prior research within the UK Biobank cohort. Second, the annual concentrations of air pollutants in the UK were relatively low, preventing us from exploring the impact of higher concentrations of air pollutant exposures on GERD. Additional research is warranted in developing countries with higher ambient air pollution levels. Third, as the UK Biobank only includes participants aged 40–69 years at baseline, our results primarily reflect associations in middle-aged and older adults and may not be fully generalizable to younger populations.

## 5 Conclusion

In conclusion, this study demonstrated that air pollution may serve as an ‌unignorable environmental risk factor for GERD. Our findings indicated that long-term exposure to both particulate matter (PM_10_, PM_2.5–10_, and PM_2.5_) and gaseous pollutants (NO_X_, NO_2_, and NO) may increase the incidence risk of GERD, particularly GERD without esophagitis rather than GERD with esophagitis. Considering the hazardous impact of air pollution, our research suggested the necessity of reducing emissions and restricting the air pollutants standards to alleviate the disease burden of GERD.

## Data Availability

The data used in this study were obtained from the UK Biobank (https://www.ukbiobank.ac.uk, application number: 99732). The authors do not have the rights to share the dataset directly.

## Footnotes

^1^ www.dagitty.net
