# Supplementary material for: Air pollution increases gastroesophageal reflux disease risk: evidence from a prospective cohort study
Source: Front Public Health. 2025 Aug 6;13:1620411. doi: 10.3389/fpubh.2025.1620411 (PMC12364854; doi:10.3389/fpubh.2025.1620411)
Supplement: Supplementary file 1 [file Data_Sheet_1.docx]

**Supplementary material**

**Supplementary Methods**

BMI was calculated as weight in kilograms divided by the square of height in meters (kg/m²). Ethnicity was categorized as White, Mixed, Asian or Asian British, Black or Black British, and Others. Physical activity was classified into low, moderate, and high levels based on the International Physical Activity Questionnaire (IPAQ) ^[1]^. Dietary habits were assessed using criteria for a healthy diet as reported in a previous UK Biobank study ^[2]^. A healthy diet was defined by increased consumption of fruits, vegetables, and fish as well as decreased intake of processed and red meats (fruit and vegetable intake: > 4.5 servings per week; fish intake: > 2 servings per week; processed meat intake: ≤ 2 servings per week; red meat intake: ≤ 5 servings per week). A healthy diet was categorized as meeting at least two of these criteria; otherwise, it was considered unhealthy. Education level was dichotomized as high (college or university degree) and low (high school or below). Smoking status and alcohol consumption were categorized as never, previous, and current. The TDI, a composite measure incorporating unemployment, household overcrowding, non-car ownership, and non-home ownership, was used to assess socioeconomic status (SES) ^[3]^, with a higher index indicating lower SES. Mental health disorder (Yes, No) was determined based on responses to the touchscreen question: “Have you ever seen a psychiatrist for nerves, anxiety, tension, or depression?”

In the DAG framework, age, sex, ethnicity, education level, Townsend Deprivation Index (TDI), and assessment centers were identified as confounders because individuals with different levels of these characteristics are likely to experience different levels of air pollution exposure and also have different risks of developing GERD. These confounders are therefore adjusting for them is essential to reduce confounding bias in the association estimates. In contrast, variables such as BMI, smoking status, alcohol consumption, physical activity, dietary habits, and mental health disorders are primarily established risk factors for GERD but are not directly associated with air pollution exposure based on existing epidemiological evidence. Therefore, these variables were not adjusted in the primary analysis but were incorporated into sensitivity analyses to assess the robustness of our findings.

References:

[1] Cleland C, Ferguson S, Ellis G, et al. Validity of the International Physical Activity Questionnaire (IPAQ) for assessing moderate-to-vigorous physical activity and sedentary behaviour of older adults in the United Kingdom [J]. BMC Med Res Methodol, 2018, 18(1): 176.

[2] Rutten-Jacobs L C, Larsson S C, Malik R, et al. Genetic risk, incident stroke, and the benefits of adhering to a healthy lifestyle: cohort study of 306 473 UK Biobank participants [J]. Bmj, 2018, 363: k4168.

[3] Blane D, Townsend P, Phillimore P, et al. Health and Deprivation: Inequality and the North [J]. Br J Sociol, 1987, 40: 344.


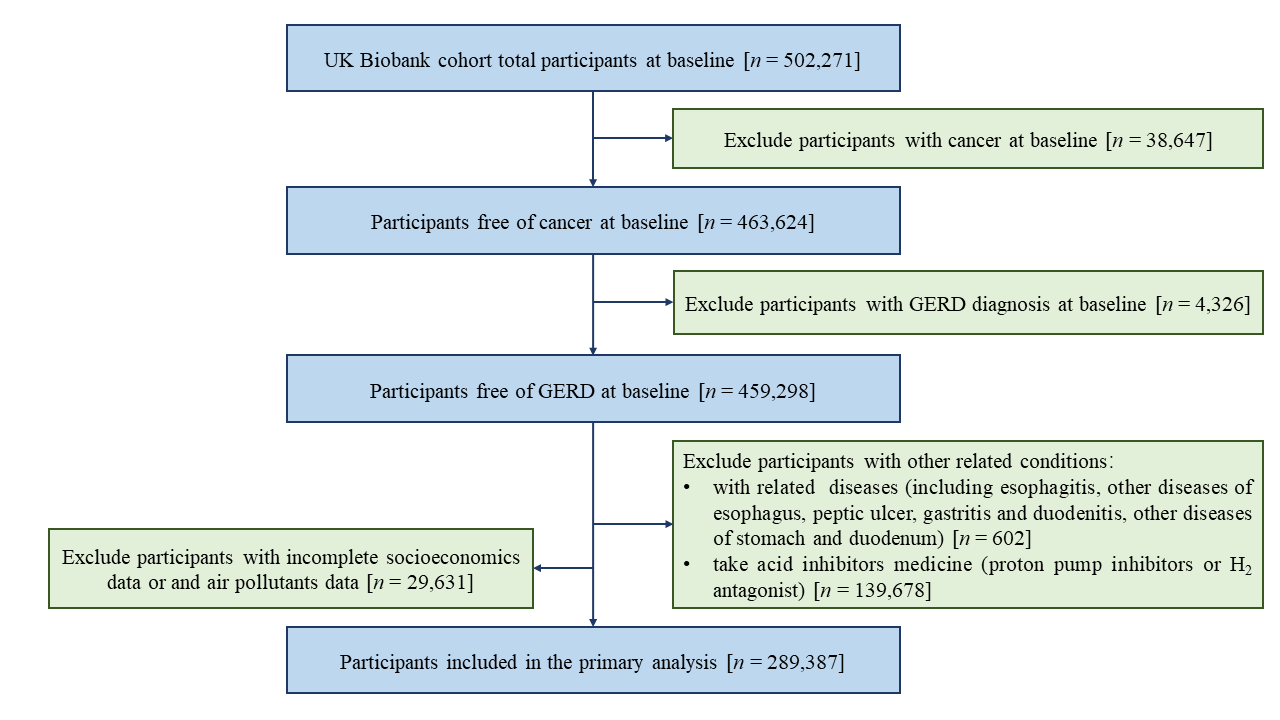


Figure S1 Flowchart of participants included in the study

Abbreviation: GERD, gastroesophageal reflux disease.


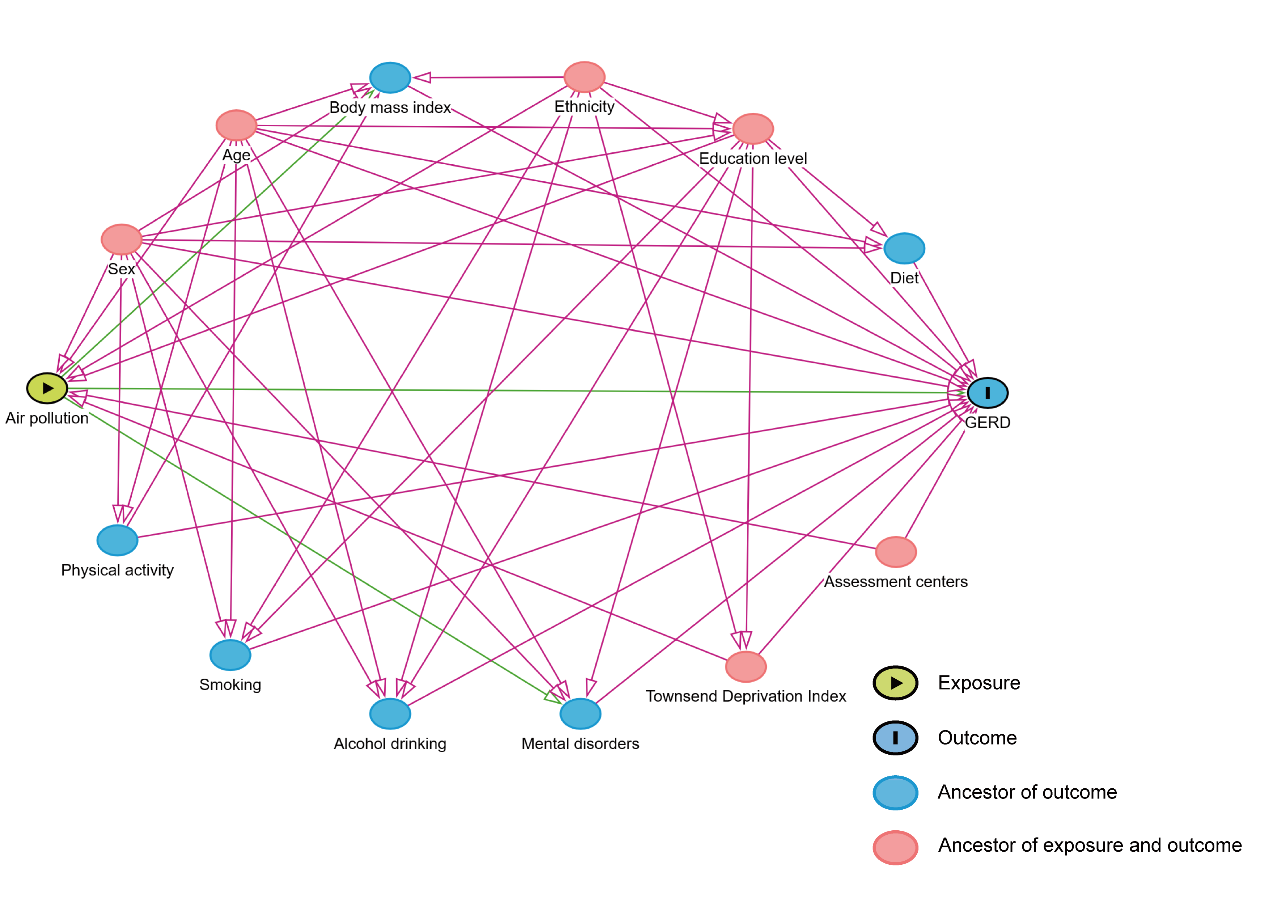


Figure S2 Directed Acyclic Graph for the association between air pollution and GERD


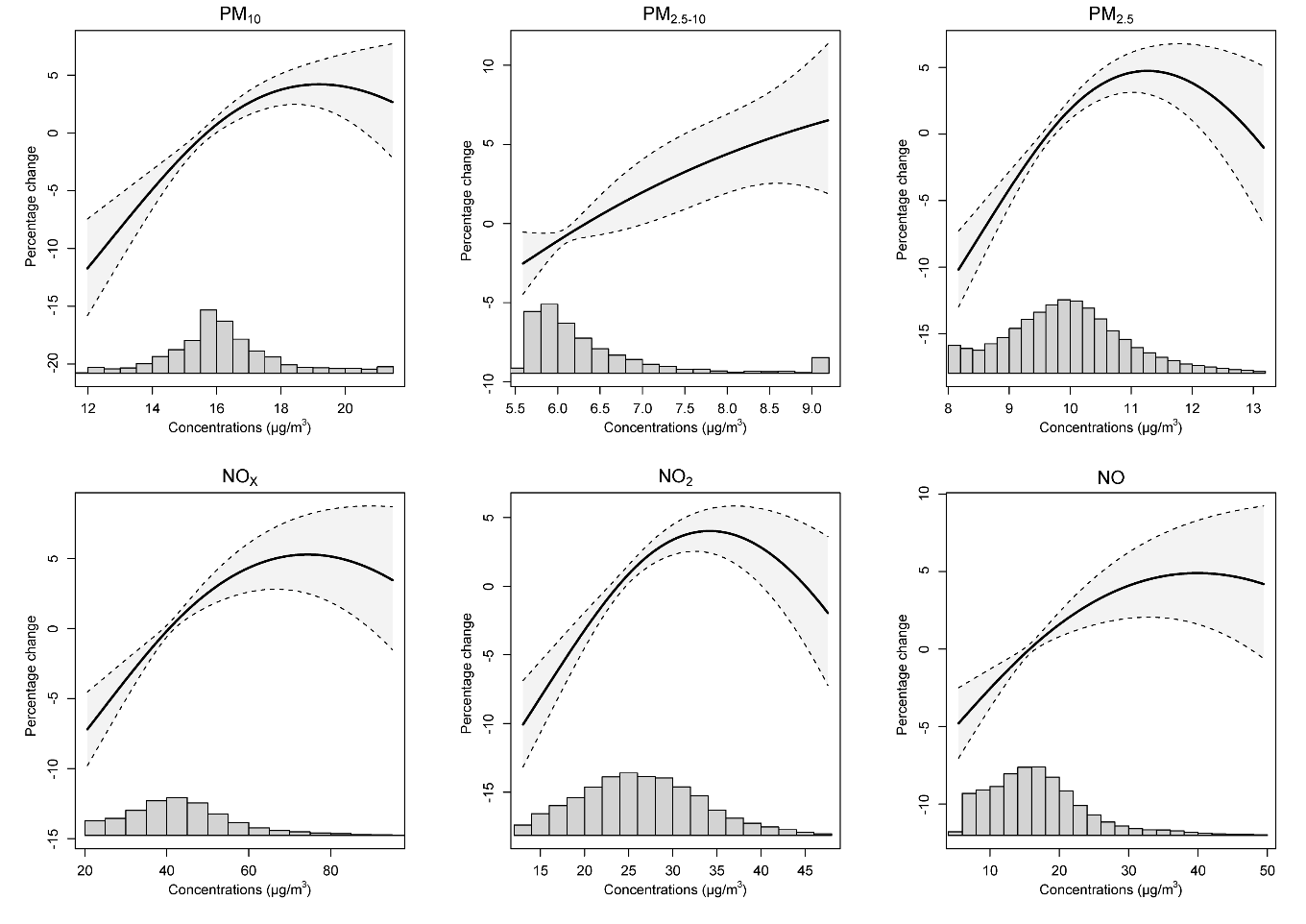


Figure S3 The exposure and response curves of long-term ambient air pollutant exposures and the percentage change of GERD without esophagitis (K21.9).

Note: the model was adjusted for age, sex, ethnicity, education level, Townsend deprivation index, and assessment centers.

Abbreviation: PM_10_, inhalable particulate matter; PM_2.5-10_, coarse particulate matter; PM_2.5_, fine particulate matter; NO_X_, nitrogen oxides; NO_2_, nitrogen dioxide; NO, nitric oxide; GERD, gastroesophageal reflux disease.


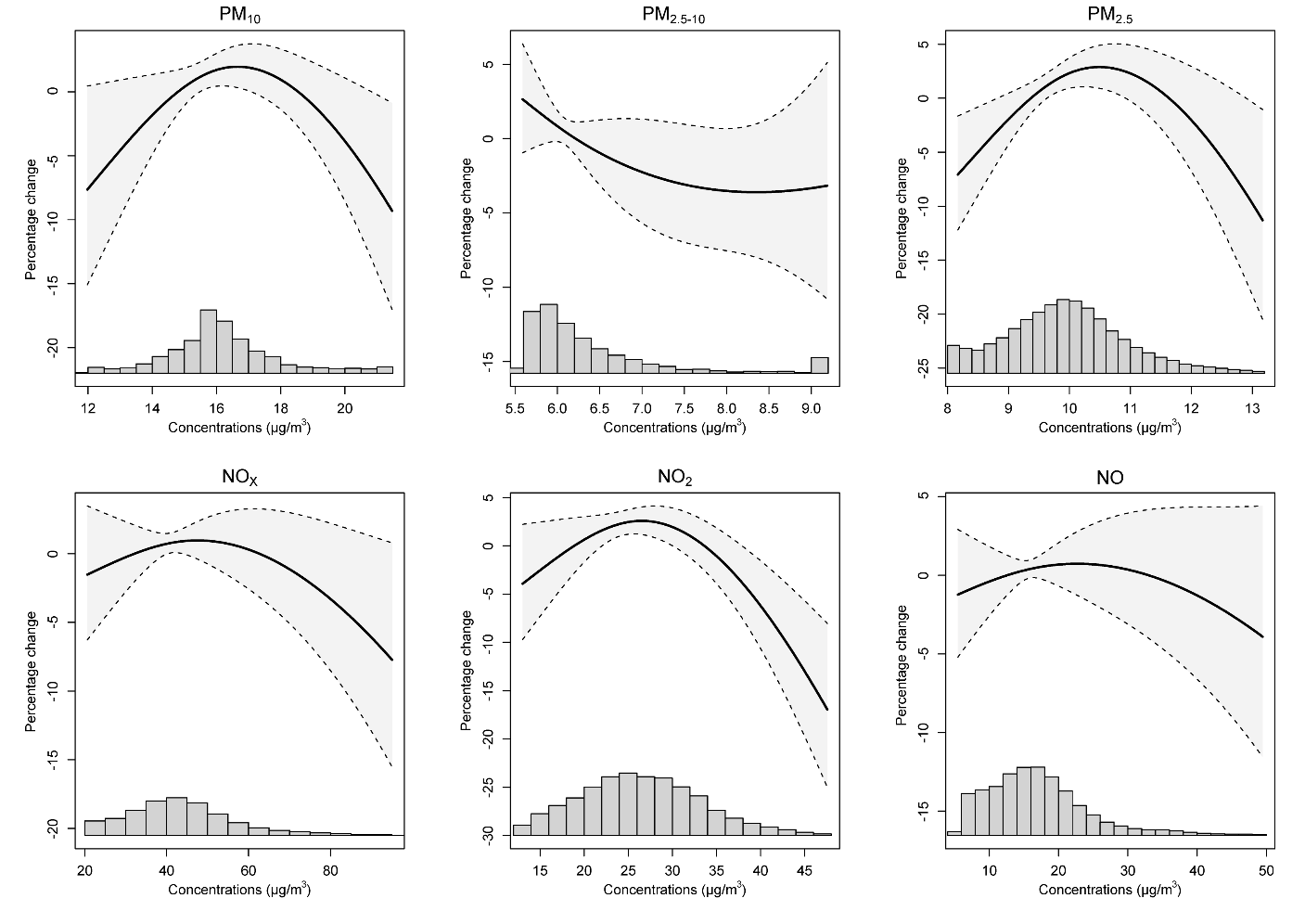


Figure S4 The exposure and response curves of long-term ambient air pollutant exposures and the percentage change of GERD with esophagitis (K21.0)

Note: the model was adjusted for age, sex, ethnicity, education level, Townsend deprivation index, and assessment centers.

Abbreviation: PM_10_, inhalable particulate matter; PM_2.5-10_, coarse particulate matter; PM_2.5_, fine particulate matter; NO_X_, nitrogen oxides; NO_2_, nitrogen dioxide; NO, nitric oxide; GERD, gastroesophageal reflux disease.

**Table S1** The Spearman correlations between air pollutants

| **Air pollutants** | **PM_10_** | **PM_2.5-10_** | **PM_2.5_** | **NO_X_** | **NO_2_** | **NO** |
| --- | --- | --- | --- | --- | --- | --- |
| **PM_10_** | 1.00 | 0.78 | 0.57 | 0.56 | 0.78 | 0.47 |
| **PM_2.5-10_** | 0.78 | 1.00 | 0.27 | 0.44 | 1.00 | 0.24 |
| **PM_2.5_** | 0.57 | 0.27 | 1.00 | 0.61 | 0.27 | 0.75 |
| **NO_X_** | 0.56 | 0.44 | 0.61 | 1.00 | 0.44 | 0.47 |
| **NO_2_** | 0.78 | 1.00 | 0.27 | 0.44 | 1.00 | 0.24 |
| **NO** | 0.47 | 0.24 | 0.75 | 0.47 | 0.24 | 1.00 |

Abbreviation: PM_10_, inhalable particulate matter; PM_2.5-10_, coarse particulate matter; PM_2.5_, fine particulate matter; NO_X_, nitrogen oxides; NO_2_, nitrogen dioxide; NO, nitric oxide.

**Table S2** Subgroup analyses of percentage change (%) in K21 incidence risk associated with each IQR in air pollutants

| Subgroup | Particulate matter | | | Gaseous pollutants | | |
| --- | --- | --- | --- | --- | --- | --- |
|  | PM_10_ | PM_2.5-10_ | PM_2.5_ | NO_X_ | NO_2_ | NO |
| Sex |  |  |  |  |  |  |
| Male | 1.11 (-0.51, 2.75) | 0.81 (-0.69, 2.33) | 1.88 (-0.41, 4.21) | **0.31 (-1.98, 2.66)** | **0.40 (-2.23, 3.09)** | **0.19 (-1.85, 2.27)** |
| Female | 2.06 (0.56, 3.58) | 1.25 (-0.11, 2.63) | 4.09 (1.94, 6.27) | **3.53 (1.38, 5.73)** | **4.40 (1.68, 7.19)** | **3.24 (1.33, 5.17)** |
| Age |  |  |  |  |  |  |
| < 60 | **3.47 (1.76, 5.21)** | 2.23 (0.69, 3.79) | **5.42 (3.02, 7.87)** | **3.86 (1.53, 6.24)** | **4.23 (1.39, 7.14)** | **3.62 (1.52, 5.76)** |
| ≥60 | **0.69 (-0.73, 2.12)** | 0.44 (-0.87, 1.78) | **1.17 (-0.86, 3.24)** | **0.43 (-1.36, 2.25)** | **0.03 (-2.31, 2.43)** | **0.60 (-0.98, 2.21)** |
| BMI |  |  |  |  |  |  |
| <25 | 2.66 (0.31, 5.06) | 1.73 (-0.41, 3.91) | 2.18 (-1.10, 5.57) | **0.14 (-2.91, 3.30)** | **0.17 (-3.48, 3.94)** | **0.11 (-2.76, 3.06)** |
| ≥25 | 1.10 (-0.2, 2.41) | 0.60 (-0.58, 1.80) | 3.86 (1.99, 5.76) | **4.01 (2.01, 6.04)** | **4.82 (2.44, 7.26)** | **3.69 (1.80, 5.61)** |
| Ethnicity |  |  |  |  |  |  |
| White | **2.02 (0.82, 3.24)** | 1.26 (0.17, 2.36) | **4.63 (2.89, 6.40)** | **3.06 (1.37, 4.78)** | **3.11 (1.06, 5.21)** | **3.28 (1.72, 4.86)** |
| Others | **-1.96 (-5.61, 1.83)** | -1.41 (-4.72, 2.01) | **-2.81 (-7.48, 2.09)** | **-3.28 (-7.39, 1.01)** | **-3.40 (-8.88, 2.41)** | **-3.06 (-6.69, 0.71)** |
| Education level |  |  |  |  |  |  |
| Low | 1.37 (0.08, 2.68) | 0.76 (-0.40, 1.93) | 3.01 (1.16, 4.89) | 1.28 (-0.49, 3.09) | 1.15 (-1.04, 3.40) | 1.64 (0.02, 3.28) |
| High | 1.34 (-1.07, 3.80) | 0.24 (-2.01, 2.54) | 4.21 (0.82, 7.71) | 4.01 (0.72, 7.40) | 5.03 (1.11, 9.10) | 3.34 (0.28, 6.50) |
| TDI |  |  |  |  |  |  |
| Low SES | **-0.43 (-2.08, 1.25)** | -0.09 (-1.59, 1.44) | **-1.71 (-3.73, 0.36)** | **-2.82 (-4.70, -0.91)** | **-4.09 (-6.45, -1.67)** | **-1.35 (-3.06, 0.38)** |
| High SES | **3.31 (1.73, 4.92)** | 1.86 (0.44, 3.30) | **8.10 (5.35, 10.92)** | **8.23 (5.38, 11.16)** | **7.73 (4.49, 11.07)** | **7.45 (4.82, 10.14)** |
| Assessment centers |  |  |  |  |  |  |
| England | 1.94 (0.76, 3.13) | **1.30 (0.23, 2.38)** | 3.34 (1.68, 5.03) | 1.92 (0.33, 3.54) | 1.76 (-0.19, 3.75) | 2.13 (0.67, 3.62) |
| Others | -2.54 (-7.11, 2.26) | **-4.15 (-8.32, 0.2)** | 8.68 (1.38, 16.51) | 6.55 (-1.12, 14.82) | 6.36 (-2.94, 16.56) | 7.64 (0.62, 15.14) |

Note: Results are expressed as the percentage change (%) in risk, and percentage signs (%) are omitted for clarity. Bolded values indicate statistically significant between-subgroup differences (*P* for difference < 0.05). The interquartile range (IQR) values for PM_10_, PM_2.5-10_, PM_2.5_, NO_X_, NO_2,_ and NO were 1.75μg/m³, 0.79μg/m³, 1.27μg/m³, 16.43μg/m³, 9.73μg/m³, and 9.01μg/m³, respectively. Education level was dichotomized as high (college or university degree) and low (high school or below). High SES was defined as Townsend Deprivation Index < -2.17 (median value) while low SES was defined as Townsend Deprivation Index ≥ -2.17.

Abbreviation: IQR, interquartile range; PM_10_, inhalable particulate matter; PM_2.5-10_, coarse particulate matter; PM_2.5_, fine particulate matter; NO_X_, nitrogen oxides; NO_2_, nitrogen dioxide; NO, nitric oxide; K21, gastroesophageal reflux disease (GERD); K21.9, GERD without esophagitis; K21.0, GERD with esophagitis; BMI, body mass index; TDI, Townsend Deprivation Index; SES, socioeconomic status.

**Table S3** Subgroup analyses of percentage change (%) in K21.9 incidence risk associated with each IQR in air pollutants

| Subgroup | Particulate matter | | | Gaseous pollutants | | |
| --- | --- | --- | --- | --- | --- | --- |
|  | PM_10_ | PM_2.5-10_ | PM_2.5_ | NO_X_ | NO_2_ | NO |
| Sex |  |  |  |  |  |  |
| Male | 1.84 (-0.02, 3.74) | 1.46 (-0.23, 3.19) | 2.75 (0.23, 5.33) | **0.36 (-2.14, 2.92)** | **0.33 (-2.95, 3.72)** | **0.37 (-1.55, 2.32)** |
| Female | 2.96 (1.41, 4.54) | 2.26 (0.84, 3.7) | 5.22 (2.95, 7.53) | **4.30 (2.12, 6.52)** | **4.91 (2.22, 7.67)** | **4.15 (2.15, 6.18)** |
| Age |  |  |  |  |  |  |
| < 60 | **4.63 (2.81, 6.47)** | **3.37 (1.72, 5.05)** | **7.51 (4.82, 10.26)** | 4.96 (2.44, 7.54) | **6.81 (3.63, 10.08)** | 4.82 (2.54, 7.16) |
| ≥60 | **0.89 (-0.70, 2.5)** | **0.8 (-0.64, 2.27)** | **2.76 (0.46, 5.12)** | 2.33 (0.14, 4.56) | **2.08 (-0.58, 4.82)** | 2.34 (0.31, 4.41) |
| BMI |  |  |  |  |  |  |
| <25 | 4.14 (1.55, 6.8) | 3.25 (0.91, 5.64) | 3.34 (-0.3, 7.12) | **1.96 (0.25, 3.7)** | **1.63 (-0.78, 4.10)** | **1.25 (-1.38, 3.96)** |
| ≥25 | 1.7 (0.33, 3.08) | 1.29 (0.05, 2.55) | 5.06 (3.06, 7.1) | **5.6 (3.37, 7.87)** | **5.99 (3.03, 9.04)** | **5.27 (3.07, 7.51)** |
| Ethnicity |  |  |  |  |  |  |
| White | **2.82 (1.58, 4.08)** | 2.06 (0.96, 3.18) | 5.59 (3.72, 7.50) | **4.14 (2.38, 5.93)** | 4.62 (2.5, 6.78) | **4.11 (2.47, 5.78)** |
| Others | **-1.98 (-6.05, 2.25)** | -1.33 (-5.44, 2.97) | -0.11 (-5.39, 5.46) | **-1.04 (-5.78, 3.93)** | 0.86 (-5.32, 7.44) | **-1.92 (-5.99, 2.33)** |
| Education level |  |  |  |  |  |  |
| Low | 1.92 (0.53, 3.33) | 1.38 (0.13, 2.64) | 4.57 (2.55, 6.63) | 3.06 (1.11, 5.03) | 3.78 (1.36, 6.26) | 2.98 (1.22, 4.77) |
| High | 2.96 (0.3, 5.69) | 2.13 (-0.33, 4.65) | 4.32 (0.61, 8.16) | 4.29 (0.69, 8.02) | 5.69 (1.39, 10.17) | 3.78 (0.41, 7.26) |
| TDI |  |  |  |  |  |  |
| Low SES | **0.16 (-1.64, 1.98)** | 0.93 (-0.7, 2.58) | **-1.05 (-3.25, 1.21)** | **-1.65 (-3.69, 0.44)** | **-2.79 (-5.37, -0.14)** | **-0.45 (-2.3, 1.44)** |
| High SES | **4.00 (2.28, 5.75)** | 2.65 (1.11, 4.21) | **9.7 (6.68, 12.81)** | **10.00 (6.86, 13.22)** | **10.51 (6.92, 14.22)** | **8.82 (5.94, 11.78)** |
| Assessment centers |  |  |  |  |  |  |
| England | **2.88 (1.6, 4.18)** | **2.31 (1.16, 3.48)** | 4.56 (2.73, 6.41) | 3.37 (1.62, 5.14) | 3.85 (1.7, 6.05) | 3.34 (1.74, 4.97) |
| Others | **-2.48 (-7.18, 2.45)** | **-4.58 (-8.86, -0.1)** | 9.58 (2.05, 17.67) | 6.39 (-1.45, 14.85) | 8.8 (-0.93, 19.48) | 6.56 (-0.58, 14.21) |

Note: Results are expressed as the percentage change (%) in risk, and percentage signs (%) are omitted for clarity. Bolded values indicate statistically significant between-subgroup differences (*P* for difference < 0.05). The interquartile range (IQR) values for PM_10_, PM_2.5-10_, PM_2.5_, NO_X_, NO_2,_ and NO were 1.75μg/m³, 0.79μg/m³, 1.27μg/m³, 16.43μg/m³, 9.73μg/m³, and 9.01μg/m³, respectively. Education level was dichotomized as high (college or university degree) and low (high school or below). High SES was defined as Townsend Deprivation Index < -2.17 (median value) while low SES was defined as Townsend Deprivation Index ≥ -2.17.

Abbreviation: IQR, interquartile range; PM_10_, inhalable particulate matter; PM_2.5-10_, coarse particulate matter; PM_2.5_, fine particulate matter; NO_X_, nitrogen oxides; NO_2_, nitrogen dioxide; NO, nitric oxide; K21, gastroesophageal reflux disease (GERD); K21.9, GERD without esophagitis; K21.0, GERD with esophagitis; BMI, body mass index; TDI, Townsend Deprivation Index; SES, socioeconomic status.

**Table S4** The results of sensitivity analyses of the associations between air pollutants and GERD and the subtype

| **Air pollutants** | | **Particulate matter** | | | **Gaseous pollutants** | | |
| --- | --- | --- | --- | --- | --- | --- | --- |
|  |  | **PM_10_** | **PM_2.5-10_** | **PM_2.5_** | **NO_X_** | **NO_2_** | **NO** |
| Main model: adjusted by age, sex, ethnicity, education level, Townsend Deprivation Index, and assessment centers | | | | | | | |
| K21 | | 1.69 (0.58, 2.81) | 1.29 (0.29, 2.29) | 3.57 (1.97, 5.19) | 2.08 (0.56, 3.63) | 1.93 (0.06, 3.83) | 2.28 (0.88, 3.70) |
| K21.9 | | 2.52 (1.31, 3.73) | 1.89 (0.80, 2.99) | 4.78 (3.03, 6.56) | 3.46 (1.80, 5.15) | 4.00 (1.94, 6.10) | 3.38 (1.86, 4.93) |
| K21.0 | | -0.30 (-2.39, 1.83) | -1.40 (-3.33, 0.56) | 1.18 (-1.78, 4.23) | -0.42 (-3.25, 2.49) | -2.26 (-5.68, 1.29) | 0.50 (-2.11, 3.17) |
| Sensitivity analysis 1: introduced all covariates (adding BMI, smoking status, alcohol drinking, diet, physical activity, and mental disorders) | | | | | | | |
| K21 | | 1.22 (0.11, 2.34) | 1.10 (0.10, 2.10) | 2.65 (0.79, 4.54) | 1.56 (0.04, 3.11) | 0.96 (-1.21, 3.18) | 1.69 (0.05, 3.36) |
| K21.9 | | 1.79 (0.38, 3.22) | 1.58 (0.29, 2.88) | 3.62 (1.58, 5.69) | 2.31 (0.36, 4.30) | 2.66 (0.27, 5.11) | 2.63 (0.83, 4.45) |
| K21.0 | | -0.45 (-3.12, 2.29) | -1.28 (-3.75, 1.25) | 1.03 (-2.75, 4.96) | -0.41 (-4.05, 3.37) | -0.63 (-5.08, 4.03) | -0.68 (-4.00, 2.77) |
| Sensitivity analysis 2: excluded participants whose GERD diagnosis occurred in the 1/2/3 years of follow-up | | | | | | | |
| K21 | 1 year | 1.64 (0.51, 2.78) | 1.26 (0.25, 2.28) | 3.56 (1.92, 5.23) | 1.89 (0.34, 3.47) | 1.84 (-0.07, 3.77) | 2.07 (0.64, 3.51) |
|  | 2 years | 1.44 (0.29, 2.60) | 1.13 (0.10, 2.17) | 3.22 (1.55, 4.92) | 1.77 (0.18, 3.38) | 1.68 (-0.26, 3.66) | 1.94 (0.48, 3.42) |
|  | 3 years | 1.36 (0.18, 2.55) | 1.11 (0.05, 2.18) | 2.75 (1.04, 4.49) | 1.65 (0.03, 3.30) | 1.56 (-0.43, 3.59) | 1.69 (0.20, 3.20) |
| K21.9 | 1 year | 2.41 (1.19, 3.65) | 1.80 (0.70, 2.92) | 4.75 (2.98, 6.55) | 3.27 (1.58, 4.98) | 3.80 (1.72, 5.93) | 3.23 (1.68, 4.80) |
|  | 2 years | 2.14 (0.90, 3.40) | 1.68 (0.56, 2.82) | 4.41 (2.61, 6.24) | 3.13 (1.41, 4.87) | 3.61 (1.49, 5.77) | 3.13 (1.56, 4.73) |
|  | 3 years | 1.98 (0.71, 3.26) | 1.59 (0.44, 2.75) | 3.93 (2.09, 5.82) | 2.96 (1.20, 4.74) | 3.32 (1.16, 5.53) | 3.03 (1.42, 4.66) |
| K21.0 | 1 year | -0.24 (-2.39, 1.96) | -1.19 (-3.17, 0.83) | 1.05 (-2.00, 4.19) | -0.62 (-3.53, 2.38) | -2.06 (-5.59, 1.61) | 0.09 (-2.59, 2.84) |
|  | 2 years | -0.17 (-2.39, 2.10) | -1.02 (-3.06, 1.06) | 0.72 (-2.41, 3.95) | -0.88 (-3.87, 2.21) | -2.34 (-5.97, 1.43) | -0.31 (-3.07, 2.53) |
|  | 3 years | -0.30 (-2.39, 1.83) | -1.40 (-3.33, 0.56) | 1.18 (-1.78, 4.23) | -0.42 (-3.25, 2.49) | -2.26 (-5.68, 1.29) | 0.50 (-2.11, 3.17) |
| Sensitivity analysis 3: restricted the analysis to participants who lived in their current address for at least 10 years | | | | | | | |
| K21 | | 1.48 (0.18, 2.79) | 1.26 (0.08, 2.44) | 4.02 (2.10, 5.97) | 2.47 (0.65, 4.32) | 2.71 (0.47, 5.00) | 2.79 (1.11, 4.51) |
| K21.9 | | 2.44 (1.02, 3.87) | 1.81 (0.52, 3.11) | 5.3 (3.20, 7.43) | 3.82 (1.83, 5.85) | 4.77 (2.31, 7.29) | 3.84 (2.00, 5.70) |
| K21.0 | | -1.28 (-3.72, 1.21) | -1.64 (-3.92, 0.69) | 1.16 (-2.37, 4.81) | -0.54 (-3.90, 2.94) | -1.69 (-5.77, 2.57) | 0.32 (-2.79, 3.54) |

Note: Results are expressed as the percentage change (%) in risk, and percentage signs (%) are omitted for clarity.

Abbreviation: PM_10_, inhalable particulate matter; PM_2.5-10_, coarse particulate matter; PM_2.5_, fine particulate matter; NO_X_, nitrogen oxides; NO_2_, nitrogen dioxide; NO, nitric oxide; K21, gastroesophageal reflux disease (GERD); K21.9, GERD without esophagitis; K21.0, GERD with esophagitis.

**Table S5** The results of the associations between long-term air pollutant exposures and incidence risks of traffic accidents (negative outcome control).

|  | Percentage change (95%CI) | *P* value |
| --- | --- | --- |
| Particulate matter |  |  |
| PM_10_ | -2.27 (-4.84, 0.37) | 0.09 |
| PM_2.510_ | -0.48 (-2.87, 1.98) | 0.70 |
| PM_2.5_ | -3.28 (-6.8, 0.41) | 0.08 |
| Gaseous pollutants |  |  |
| NO_X_ | -2.15 (-5.65, 1.49) | 0.24 |
| NO_2_ | -3.59 (-7.79, 0.81) | 0.11 |
| NO | -1.45 (-4.69, 1.90) | 0.39 |

Note: Results are expressed as the percentage change (%) in risk, and percentage signs (%) are omitted for clarity. Transport accidents (ICD-10: V01-V99) were chosen as the negative outcome control, and the model was adjusted for age, sex, ethnicity, education level, Townsend Deprivation Index, and assessment centers.

Abbreviation: PM_10_, inhalable particulate matter; PM_2.5-10_, coarse particulate matter; PM_2.5_, fine particulate matter; NO_X_, nitrogen oxides; NO_2_, nitrogen dioxide; NO, nitric oxide.
